# Casualty evacuation in arctic and extreme cold environments: A paradigm shift for traumatic hypothermia management in tactical combat casualty care

**DOI:** 10.1080/22423982.2023.2196047

**Published:** 2023-05-10

**Authors:** Titus J. Rund

**Affiliations:** 207th Aviation Troop Command, Alaska Army National Guard, Joint Base Elmendorf-Richardson, AK, USA

**Keywords:** CASEVAC Ecosystem, Tactical Combat Casualty Care (TCCC), Triad of death, MARCH change to MhARCH, Casualty Protection Unit (CPU) & Casualty Collection Point (CCP), Traumatic hypothermia, Class VIII Medical Supplies & Logistics, Golden hour, Cold Soak & Thermal Cycle, Advanced resuscitative care

## Abstract

In Arctic or extreme cold environments of Alaska, trauma care is complicated by large expanses of geography and lack of forward-positioned resources. This paper presents four hypothetical vignettes highlighting austere cold medical priorities: (1) traumatic hypothermia management as part of Tactical Combat Casualty Care (TCCC) is clinically and tactically important and hypothermia needs to be reprioritized in the MARCH algorithm to MhARCH; (2) at present it is unknown which TCCC recommended medical equipment/supplies will function as designed in the extreme cold; (3) ensuring advanced resuscitative care measures are available serves as a temporal bridge until casualties can receive damage control resuscitation (DCR); and (4) current systems for managing traumatic hypothermia in TCCC and casualty evacuation (CASEVAC) are insufficient. In conclusion, numerous assessments recognise the DoD’s current solutions for employing medical forces in Arctic operations are not optimally postured to save lives. There should be a joint standard for fielding an arctic supplement to current medical equipment sets. A new way of thinking in terms of an “ecosystem” approach of immediate casualty protection and movement in CASEVAC doctrine is needed to optimise these “Golden Minutes.”

## Introduction

Tactical Combat Casualty Care (TCCC) has advanced significantly over the last 20 years of Counter Insurgency (COIN) operations and has led to the highest level of survivability in the history of combat medicine [1,2]. In the 14 years since Secretary of Defense Robert Gates ordered that an urgent surgical casualty receive damage control surgery within the “Golden Hour,” the Department of Defense (DoD) has experienced the lowest level of mortality in its history. As we transition from COIN to new doctrinal paradigms for future conflict numerous articles are being written questioning the feasibility of delivering casualties for damage control surgery within the Golden Hour [2,3]. This may be due to expeditionary, contingency, small-scale combat operations, large-scale combat operations, or simply due to the large expanse of geography and the lack of forward-positioned resources. In operational terms, this is the “tyranny of distance and time,” an important planning factor that influences operational risk assessments and influences decisions on resource allocation to mitigate risk. In Alaska, the tyranny of distance and time is particularly significant as the state is sparsely populated, road infrastructure is limited and medical infrastructure outside of the main cities lack surgical coverage that medical planners could use in contingency operations.

Understanding the Arctic as a unique operations environment, the DoD published an Arctic Strategy in 2019 placing a renewed focus on medical operations in Arctic or extreme cold environments (AoECE). Each military branch has now published their own Arctic Strategy and this is a trigger for medical planners to assess current Doctrine, Organization, Training, Materiel, Leadership and Education, Personnel, Facilities and Policy (DOTMLPF-P). The United States Air Force Air Combatant Command (ACC) Surgeon (SG) developed a DOTmLPF-P Change Recommendation (DCR) after meeting with arctic medicine subject matter experts during a high performance team meeting. This DCR highlighted that current solutions for employing medical forces in AoECE are not optimally postured to save lives and ease suffering. Note, in a DCR, materiel solutions (i.e. those items that require acquisition such as equipment and supplies) are limited to execution year funding and not future year funding [4]. Of particular interest for this paper is the care of traumatically injured casualties below the level of a NATO Role 1 Battalion Aid Station (BAS) where traumatic hypothermia is difficult to manage. This paper will lay out the need for further research and development as well as the need for a paradigm shift in how we approach TCCC, Advanced Resuscitative Care (ARC), Casualty Evacuation (CASEVAC), Medical Evacuation (MEDEVAC) and the medical logistics needed to support medical operation in this arctic or extreme cold environment. This article is written in the specific context of the Alaskan AoECE due to its limited infrastructure, road systems and small population relative to that of other NATO and Scandinavian partners.

Hypothetical scenarios faced by military servicemembers in Alaska are presented below as a framework for identifying key capability gaps of Tactical Combat Casualty Care (TCCC) in AoECE. The goal is to bring awareness in the hopes that it will stimulate research, technological innovation, jumpstart development of new equipment and training, and generate requirements that can be actioned by the DoD. The management of traumatic hypothermia will take a central focus in this discussion as the extreme cold weather environment can cause a traumatic injury to go from being treatable to life-threatening in a matter of minutes. The last scenario presents a proposal for a doctrinally aligned change in how TCCC may be optimised in Arctic or extreme cold environments.

## Methods

Four scenarios were created to illustrate current high priority medical challenges in AoECE. These are based on the author’s personal experience as a physician assigned to the Alaska Army National Guard with responsibility for medical planning in this environment. While these are hypothetical scenarios, each one includes elements of real events and planning factors.

## Results

### Essential concept: traumatic hypothermia and the triad of death, a need for changing MARCH to MhARCH

#### Scenario 1

*A 21-year-old Soldier from Washington State is training in remote Alaska. While on a long-range patrol in flat-light conditions (low contrast visibility), he is traumatically injured when his snowmachine rolls over and he is crushed. On extrication he is observed to have an unstable pelvis. A NATO 9-Line MEDEVAC request is radioed and the ground force commander is notified that Aviation MEDEVAC is unavailable due to forecasted weather in the next six hours. The patrol medic applies the principles of Tactical Combat Casualty Care (TCCC) starting with the MARCH algorithm as trained. He applies a pelvic binder, places the casualty in an improvised hypothermia wrap and administers pain control medication. Without the ability to provide active rewarming, the casualty becomes unconscious within the hour.*

If aviation assets are unavailable, ground evacuation is limited off of the Alaskan road system. In the Alaskan AoECE, it is not unusual for rescues or evacuations to take three to five days to complete. Whether evacuation is within the “Golden Hour” or prolonged, it is the mastery of the small disciplines that take on singular importance. These small disciplines have synergistic effects which will either optimise or harm casualties and impact their outcomes on arrival for damage control surgery.

Hypothermia management of casualties is central to medical operations in AoECE. While MARCH (Massive haemorrhage, Airway, Respirations, Circulation, Hypothermia and Head Injury) is the evidence-based process a TCCC practitioner should follow in order to intervene on preventable death in a combat trauma casualty, many TCCC practitioners in Alaska are advocating for hypothermia to be redesignated as the second priority (transforming MARCH into MhARCH). There are early efforts working to codify this as a formal change in the Committee on Tactical Combat Casualty Care (CoTCCC) guidelines for AoECE [5,6].

The current hypothermia guidelines are driven by thresholds determined to be significant in “accidental hypothermia” scenarios. However, in the case of this Soldier, and of particular concern to military operational planning, new research now demonstrates that the core body temperature in “traumatic hypothermia” is a higher threshold than described in the “accidental hypothermia” populations. The Wilderness Medical Societies’ 2019 Clinical Practice Guideline (CPG) for Accidental Hypothermia defines a core body temperature of 35–37°C as “cold stress” and a core body temperature of 32–35°C as “mild hypothermia.” This CPG does discuss trauma patients as a special population in the “mild hypothermia” temperature range and recommends active rewarming [7]. A 2022 retrospective review of the DoD’s Trauma Registry (DoDTR) by Schauer et al. looked at “traumatic hypothermia” and found that a core body temperature of 36.2°C is a threshold value for worsening mortality in the combat trauma population and “current methods for pre-hospital warming are insufficient” [8]. Without appreciating the significance of this higher threshold value in the “traumatic” vs “accidental” populations, interventions may be delayed affecting mortality. Conversely, if novel solutions to arrest or preferentially reverse hypothermia can be employed sooner, a TCCC practitioner can optimise a casualty for delayed damage control resuscitation. Using 36.2°C as a sentinel signal should trigger a paradigm shift in our approach to medical operations in AoECE and further supports a shift from MARCH to MhARCH.

As we increase military operations in the AoECE, we cannot take for granted that what worked in the Global War on Terror (GWOT) will work when applied in this extreme environment. Unlike the experience in Afghanistan, Iraq and Syria, the cold exposure of the AoECE is synergistic in its effects as “hypothermia” is both an environmental exposure and a key feature of the metabolic derangement known as the “triad of death.” The “triad of death” is a physiologic/metabolic derangement where three distinct physiologic factors (hypothermia, acidosis, coagulopathy) interact and worsen the physiologic function of the other two factors. Of the three factors, traumatic hypothermia management has significant room for optimisation in the care of casualties in AoECE. Beldowicz et al. bolsters the case for optimising the management of metabolic derangements in their 2020 article “Death Ignores the Golden Hour.” This article highlights research by Eastridge et al. that found in the decade that followed 9/11, one in four casualties that died before reaching a hospital for damage control surgery (DCS) had potentially survivable injuries. These findings represent servicemembers were potentially dying of preventable death and that metabolic derangements are “easier to prevent than they are to reverse, and reversal of these conditions is easier earlier in their course” [3]. In the AoECE, this statistic is anticipated to worsen due to the interaction of the environment on traumatic hypothermia.

### Medical equipping and research

#### Scenario 2

*A 32-year-old Soldier from Colorado is on day 14 of a field exercise in an Alaskan Training Area. During a live-fire exercise he is shot in the right leg. The Combat Life Saver (CLS) removes his outer mittens for improved dexterity and immediately applies a tourniquet over the Soldier’s cold weather clothing. The tourniquet was taken from the casualty’s cold-soaked individual first aid kit (IFAK) and breaks in the −37^o^C AoECE. The CLS then grabs a tourniquet he has kept close to his body and applies it successfully. While working through the priorities of MARCH, the CLS loses motor function as his fingers quickly cool inside his contact gloves which are sweat-soaked from exertion and wet from contact with the snow. *The wind-chill on the wet contact gloves quickly induces a mild freezing injury*. The unit’s medic arrives and draws up ketamine for pain control but it freezes before it can be administered. The medic then gives a Fentanyl lollipop for pain control and they prepare the casualty for MEDAVAC while a NATO 9-Line MEDEVAC request is sent up. Due to the high-risk nature of the exercise, Aviation MEDEVAC was prepositioned and delivered the casualty to the ER for damage control surgery 1 hour and 22 minutes after initial injury.*

At present, it is unknown if the TCCC-recommended Class VIII items (medical supplies) which have served the U.S. Military so well in GWOT will function as intended in AoECE. This raises the following questions:

- Which CoTCCC recommended Class VIII supplies and medications work in the AoECE?
- Is there an exposure duration or an absolute temperature threshold at which a Class VIII item or medication should be considered to be non-serviceable?
- Does thermal cycling have an adverse impact on Class VIII supplies?
- What are the freezing temperatures of CoTCCC recommended medications and if warmed or thermally cycled will they lose therapeutic efficacy?
- Is there a sustainable process for off-body carry of temperature-sensitive items?
- Is there an optimal way to conduct on-body carry of temperature-sensitive items that does not interfere with a TCCC practitioner’s mobility and/or delivery of care?
- Which medical electronics are optimally suited for the following: (1) on-body carry, (2) minimal battery consumption (for low-logistic support) and (3) are robust enough to operate in the AoECE while meeting the minimum standard for prolonged care/prolonged field care?
- Are warm fresh whole blood (WFWB) transfusion kits susceptible to failure with thermal cycling? What is the cooling rate of a unit of WFWB at different ambient temperatures and is there a matrix/table (threshold ambient temperature, time from draw) or a normogram that a TCCC practitioner can use to determine if they can safely administer a unit of WFWB if a blood fluid warmer is not available forward of a NATO Role I?
- Is there an optimal way to facilitate uninterrupted IV product administration during En-Route Combat Casualty Care (ERCCC) while concurrently mitigating freezing of equipment, lines and IV products?
- Are there simple packaging or design changes for Class VIII medical supplies, equipment and medications that can help the TCCC practitioner wearing protective gloves maintain dexterity and minimise their exposure risk when providing care?

Answering these questions not only ensures that the TCCC practitioner has the appropriate Class VIII medical supplies they need but it can also help inform the development of an arctic supplement for Medical Equipment Sets (MES) for units assigned to operate in the AoECE. Additionally, this information can be used to inform the development of an authorised stockage list (ASL) for both standardising medical logistics ordering and planning for contingency operations and contingency stockpiles. This will help establish a hierarchy of controls that sets safeguards against failure in the AoECE which meets a moral obligation and collaterally impacts morale knowing that the due diligence has been done [3]. Additionally, innovation in one system spurs innovation in another. What benefits military interests will benefit civil interests in extreme, expedition and wilderness medicine as they share information and grow from each other’s research interests and outcomes [1,7,9].

### Tactical combat casualty care and advanced resuscitative care

#### Scenario 3

*A 23-year-old Soldier is participating in an airborne exercise in remote Alaska. Due to the remote nature and associated risk of the exercise a NATO Role 1 Battalion Aid Station (BAS) was established. The BAS brought two units of packed red blood cells (PRBCs) in support of TCCC Advanced Resuscitative Care (ARC) guidelines. A HH-60 M MEDEVAC helicopter in direct support of the exercise was scheduled to arrive 90 minutes after the conclusion of airborne operations for a scenario-based MEDEVAC mission. 20 minutes after airborne operations concluded, a NATO 9-line request was received for a real-world urgent surgical poly-trauma casualty who fell off a ridgeline into a deep ravine. At the time of casualty handoff, a left sided chest tube was in place, a junctional tourniquet with pelvic binder applied, and tranexamic acid (TXA), ketamine and oxygen were administered.  One unit of PRBCs was initiated for hypotension immediately prior to handoff to the critical care flight paramedic (CCFP). Due to equipment non-compatibility between the ground force and MEDEVAC blood fluid warmers, the PRBC unit had to be fully administered prior to transport further delaying the handoff.  The CCFP resupplied the ground force by providing them with one unit PRBC from their supply.  Despite conventional packaging techniques for hypothermia prevention, the casualty continued to cool en route as the cabin temperature of the aircraft equilibrated with the environment.*

Pre-mission planning in remote Alaska is vital as the geography limits mobility and resources are scarce. Delivering a casualty for damage control resuscitation can be complicated by weather and other factors so staging the appropriate medical capabilities as close to the point of injury as possible is critical to mitigate the tyranny of distance, time, and environment. In the extreme cold, addressing traumatic hypothermia starts on the ground at the point of injury, but capability gaps in keeping a casualty alive during transport should be anticipated and addressed prior to injury. The older Blackhawk model HH-60 L is equipped with an arctic heater making it possible to warm the cabin interior for better control over hypothermia management. However, the newer Blackhawk model HH-60 M being fielded in Alaska is not currently equipped with an arctic heater. The Wilderness Medical Society’s Clinical Practice Guidelines (CPG) recommend that air and ground ambulance cabin temperatures be maintained at no less than 24°C [7]. As arctic heaters are developed for the HH-60 M (and future vertical lift platforms), this is an important planning factor to consider. Any military helicopter used for MEDEVAC or CASEVAC in Alaska where temperatures routinely fall far below 24°C should be equipped with heaters that are capable of quickly heating the interior cabin to this minimum recommended temperature.

Alaska is heavily reliant on aviation because the limited road infrastructure hinders the movement of casualties using currently fielded mobility platforms. Consequently, there is a need for agile solutions to quickly maneuver and establish shelter and warmth so that advanced resuscitative measures can be supported. While these agile solutions are being developed and fielded, it is important to be deliberate in establishing a Battalion Aid Station (BAS) and multiple casualty collection points as close to the point of injury as possible. No significant TCCC interventions should occur where the casualty would be exposed to the elements as exposure would hasten hypothermia and worsen metabolic derangements.

Once the casualty is sheltered from the AoECE in a warm environment, MhARCH can be continued with a plan for advanced resuscitative measures. In the scenario above, effective operational planning ensured early blood administration was available close to the point of injury. TCCC Advanced Resuscitative Care (ARC) guidelines support early whole-blood resuscitation for casualties in haemorrhagic shock. This has the potential to improve a casualty’s survival in instances where damage control surgery cannot be initiated within the “Golden Hour.” Gurney et al.’s 2022 work highlights that prehospital transfusion when initiated within 30 minutes of injury is associated with improved survival at both 24-hours and 30-days [10]. However, the benefit of early blood administration can be compromised if given in an environment that doesn’t control for hypothermia. Schauer et al. found that the use of blood products was highest in the hypothermia population and concluded that “early, more aggressive interventions are imperative to stave off effects of hypothermia” [8]. For operational planning in AoECE, the implications are two-fold: 1) logistics of supply need to support timely availability of blood products within 30 minutes of injury, and 2) sheltering and active warming of casualties is necessary to prevent the need for more blood due to uncontrolled hypothermia. The more blood a casualty consumes, the greater the risk for mortality. April et al.’s work looking at the optimal threshold for predicting 24-hour mortality of combat trauma casualties concluded that transfusion of two units of PRBC was the threshold for 90% sensitivity and 33 units of PRBC was the threshold for 90% specificity in predicting mortality [11]. Hypothermia management in the AoECE must be optimised as close to the point of injury as possible to prevent a cascading requirement for more and more blood products.

Pre-hospital blood product transfusion relies on sourced blood products being administered via a blood fluid warmer that can deliver IV fluids at a rate up to 150 mL/min with a 38°C output temperature [12]. At present, there is anecdotal evidence but limited independent objective evidence identifying which blood fluid warmers (and their associated batteries) are optimised to meet these specified requirements in the AoECE. Due to the heavy reliance on aviation assets and prolonged flight times in Alaska, it would be beneficial to standardise blood fluid warmers and ensure that they all have an Air Worthiness Release (AWR) for use in flight. This would ensure interoperability between ground forces and aviation MEDEVAC as well as standardise logistics and supply in this resource limited environment. Blood transfusion as a component of ARC is designed to help mitigate high-risk, high-threat, variable-likelihood events for trauma-related injuries. Identifying which blood fluid warmers work in the AoECE further ensures that critical life-saving equipment is not compromised by the cold and contributing to the “triad of death.”

If a casualty in the AoECE requires ventilatory support at the point of injury, a bag-valve mask is used. This moves cold ambient air into the lungs which drops core body temperature and accelerates the development of traumatic hypothermia. Casualties who require ventilation need warm and humified air. At present there is no readily available equipment for this use in the pre-hospital environment. The DoD recognises this capability gap and recently began work on a patented design that will both warm and humidify ambient air and oxygen for a ventilated casualty [13]. In addition to warming air above ambient temperature the collateral effect is two-fold: (1) warm air will not contribute to core body cooling in the way that cold air does and (2) the oxygen-haemoglobin dissociation curve is optimised for exchange of oxygen and carbon dioxide at the alveolar-capillary membrane. This ensures that transfused blood products have maximal benefit. Additionally, other government agencies are developing a modernised charcoal-based heating system with a minimal battery requirement. This would be optimally suited for use in the AoECE where batteries deplete quickly in the cold and the power needed to recharge them may be limited. This design would have the ability to begin warming the casualty at the point of injury and can be used as an additional heat source at a casualty collection point prior to reaching a NATO Role 1 aid station [14].

### Casualty Evacuation (CASEVAC) - a proposal for a doctrinally aligned CASEVAC ecosystem from POI to patient handoff with dedicated medical evacuation (MEDEVAC) assets

#### Scenario 4

*A 24-year-old Soldier participating in an airborne exercise is carried by prevailing winds off the drop zone into rough terrain with multiple broken ribs and a femur fracture. A medic & a combat life saver (CLS) arrive within minutes and immediately begin care. While the medic conducts MhARCH, the CLS initiates aggressive hypothermia management by placing a chemical heat blanket under the casualty’s jacket. He directs fellow Soldiers to help assemble a hypothermia wrap utilizing the casualty’s sleeping bag, insulated pad, and two vapour barriers [7]. The casualty is then secured in a transport sled and prepared for movement to the casualty collection point (CCP) 500m away. The movement is stopped multiple times in order to clear snow that collects around the head, neck and upper torso. On arrival at the CCP, the medic covers the casualty with a poncho and conducts deliberate and selective exposure in order to reassess the casualty while an Arctic 10-man tent and stove are still being assembled. A NATO 9-Line for ground MEDEVAC was called, but the ambulance slid off the road and was unable to respond. Subsequently, range control calls for aviation MEDEVAC which is anticipated to arrive in 30 minutes. Despite improvised sheltering at the CCP, the casualty is observed to become unconscious as he is flown to the local hospital for resuscitation.*

The current system for hypothermia prevention has limited fielded options, requires a deliberate effort to improvise shelter, move casualties, and is not well suited to protect casualties from traumatic hypothermia. In 2022, Schauer et al. looked at the interventions used by military medics in Afghanistan, Iraq and Syria to prevent hypothermia and concluded that none of the current modalities for casualty warming are sufficient. They hypothesize that combat operations in cold weather settings would likely see a significant increase in the incidence of casualties with hypothermia [8]. In the Alaskan AoECE, patient packaging for hypothermia management requires increased insulation measures which are often improvised from a casualty’s sleeping bag, sleeping pad, as well as other items which can serve as a vapour barrier. These strategies are not optimal as the casualty is exposed while the hypothermia wrap is assembled and a decision on whether to move or shelter is made. Additionally, the traditional Ahkio tent system that a small unit/patrol would carry can take upwards of 45 to 120 minutes to set up depending on the environmental conditions and proficiency of the team [15]. Active rewarming of a casualty is traditionally performed using boiled water and heat from the Ahkio tent stove or a dedicated chemical heat blanket. In the scenario above, by the time the casualty arrives at a NATO Role 1 he has been exposed to the environment without sufficient hypothermia prevention or active rewarming measures for the duration of transport.

The capability gaps described in the scenarios above lay out a case for a paradigm shift in how TCCC and Casualty Evacuation (CASEVAC) is conducted in the AoECE. Instead of looking at individual capabilities as isolated interventions, there is a need to look at how the casualty flows through a cohesive CASEVAC ecosystem with minimal environmental exposure. The following discussion introduces a proposed CASEVAC ecosystem that is aligned with the doctrinal principles of TCCC and is designed to address the cross-section of hypothermia management from the point of injury to delivery of a casualty to a higher echelon of care.

To understand the methodology and the ecosystem’s design and function, it is first important to understand the TCCC principles of MARCH/MhARCH that are applied through the continuum of casualty evacuation (CASEVAC). CASEVAC is broken into three phases of care beginning with (1) Care Under Fire (CUF), (2) Tactical Field Care (TFC) and (3) Tactical Evacuation (TACEVAC) which moves a casualty to an ambulance exchange point (AXP) or helicopter landing zone (HLZ). CASEVAC terminates when the casualty is transferred to dedicated medical assets.

**Figure 1.**
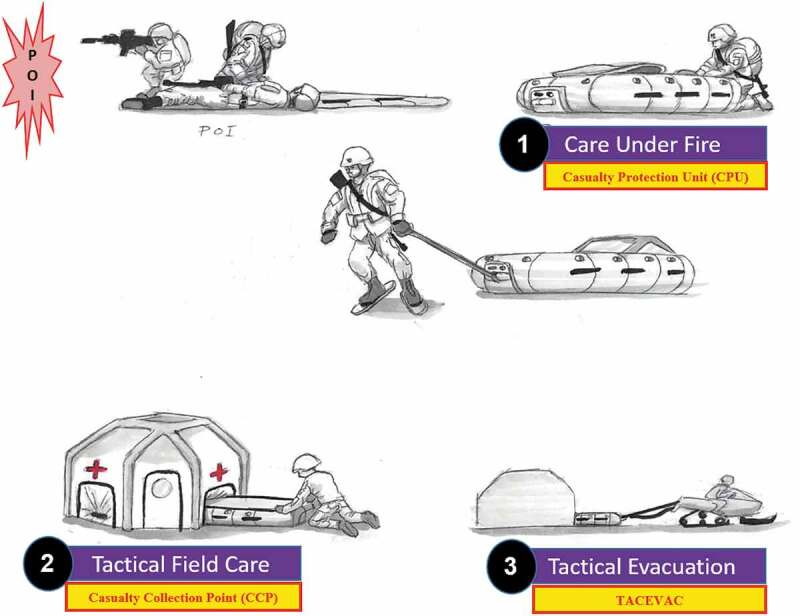
CASEVAC Ecosystem Doctrinal Alignment with Tactical Combat Casualty Care.

In the Care Under Fire (CUF) phase, the TCCC practitioner faces threats from continued enemy action and/or environmental exposure in the AoECE. Focused and expedient action to remove the casualty and protect the TCCC practitioner is necessary. The core component of this CASEVAC ecosystem design lies in the purpose-built Casualty Protection Unit (CPU) which is designed to arrest hypothermia by quickly protecting a casualty from the environment. It is waterproof, buoyant and prevents intrusion of cold air, snow or water from further cooling the casualty. Additional design features include active heating of the casualty and protection from trauma during transport. The inflatable design variants can be employed rapidly by one TCCC practitioner (see Figure 1). The AoECE has many sub-climates including littoral coast lines as well as areas which experience rapid thawing or overflow where water could otherwise cold soak a casualty in the current casualty packaging modalities.

In the Tactical Field Care (TFC) phase the complete application of a MARCH or MhARCH assessment and interventions are performed. For this to occur in the first ten minutes, casualty movement to an area that is tactically “secured” and/or environmentally “protected” must occur quickly. The second component of the CASEVAC ecosystem’s design lies in the purpose-built Casualty Collection Point (CCP). By design, the CCP is optimised to maximise functional space while minimising weight and cubic volume which allows for improved preservation of heat and is further optimised utilising thermo-reflective materials.

**Figure 2.**
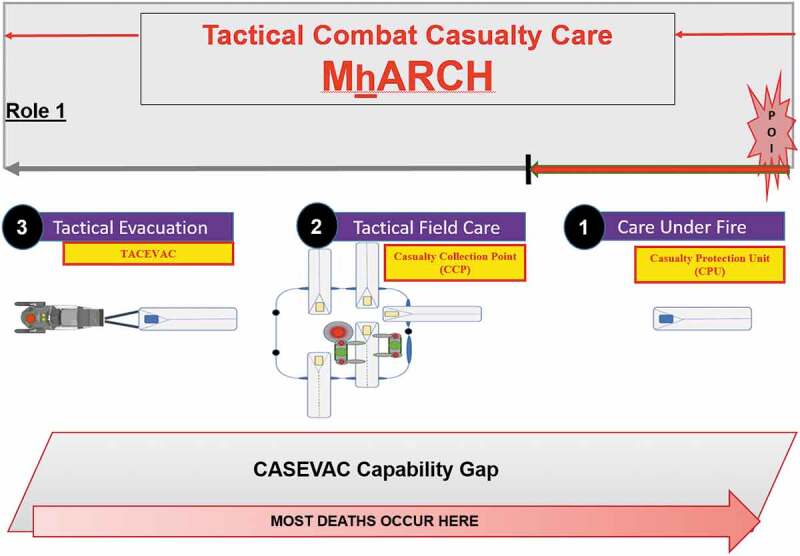
AoECE Casualty Evacuation (CASEVAC).

The CCP by design has multiple portals that allow for the passage of the CPU into the CCP (see Figure 2). The CCP provides rapid sheltering for both the casualty and the TCCC practitioner. The portals serve a multipurpose role. First, they allow the casualty within the CPU to slide directly into the CCP for expedited reassessment without exposing the casualty to the environment. Second, the portal design removes the current need to unpack the casualty from a sled or pulk and then lift the casualty into a tent. This optimises human performance by eliminating the potential for slips, falls or other injuries inherent to casualty transfer. Third, the CCP can be erected anywhere as the floor can be removed or omitted in different design variants if needed due to rocky or uneven terrain. This can be done without compromising casualty care because the CPU design distributes the casualty’s weight, serves as a treatment platform, and prevents heat loss. By utilizing multiple portal entries instead of a singular door, continuous reassessment of multiple casualties can be conducted simultaneously. When a casualty needs to be fully or partially exposed, they can be slid further into the CCP for deliberate assessment and interventions.

In the TACEVAC phase of CASEVAC, the CPU is optimised for rapid transport of a casualty. The casualty within the proposed CPU would receive a final reassessment, any necessary interventions and a casualty report without needing to be repackaged. While this is being conducted inside the CCP, the transport team would be preparing the CPU for immediate transport once handoff is complete. This prevents the undue delay associated with the current practice of repackaging, transferring, and securing the casualty into a sled or pulk. This also eliminates the associated risk for slips, falls, lifting injuries and environmental exposure. If the casualty (in a CPU) is evacuated via a snowmachine the CPU would tie into “parasitic power” and a communication system connected to the snowmachine. The design feature enables active reheating and real-time communication for continuous monitoring of a casualty’s sensorium. This allows a snowmachine driver and medic to rapidly address a casualty’s concerns and complaints as well as observe for changes in mental status.

## Conclusion

Current assessments recognise that the DoD cannot employ the full spectrum of medical care in arctic or extreme cold environments (AoECE). Outside of AoECE, the paradigm of the “Golden Hour” is already being challenged and identified to be as little as 19 to 23 minutes [16]. In this extreme environment, it can take that long to initially conduct MARCH and package a casualty for movement, and a new recommendation for changing MARCH to MhARCH to doctrinally codify this recommendation is underway by multiple military providers [5,6]. Traumatic hypothermia and its attendant interaction on the “triad of death” is a significant area of both clinical and operational concern given the new data supporting a core body temperature threshold of 36.2°C is an inflection point for mortality. Continued research is needed to identify which TCCC supplies and equipment are optimised for use in the AoECE in order to ensure that the applied interventions do not fail as a consequence of their fielding in this environment. Advancing early resuscitation with fresh whole blood and identifying which blood fluid warmers work in the AoECE further ensures that critical life-saving equipment is not compromised by the cold and contributing to the “triad of death.” This information should drive a joint standard for fielding of an arctic supplement to current medical equipment sets and assemblages. Identifying key capability gaps will help generate requirements that will continue to drive funding, research, development and future fielding of needed resources. When an hour matters, minutes count and in minutes the efforts of the disciplined application of TCCC and ARC can be blown away by the wind, wet and cold. Consequently, a new way of thinking in terms of an “ecosystem” approach of immediate casualty protection and movement as part of CASEVAC doctrine is needed to optimise these “Golden Minutes.” Military Medicine should prioritize its focus on “prehospital care because most preventable deaths occur before casualties reach combat hospitals” [17]. Furthermore, we do not want to lose the lessons learned from COIN data to the “Walker Dip.” This addresses the tendency that the lessons learned in conflict are lost in the inter-war period and have to be relearned in future conflict [1]. If we are to avoid the pitfalls of the “Walker Dip” and conserve the fighting strength, the time to innovate is now!
